# Breast enlargement associated with low dose olanzapine

**DOI:** 10.4103/0253-7613.75681

**Published:** 2011-02

**Authors:** Ashish Aggarwal, Ashish Khandelwal, Manish Jain, R.C. Jiloha

**Affiliations:** Department of Psychiatry, G.B. Pant Hospital, New Delhi, India; 1 Department of Psychiatry, Dr. Ram Manohar Lohia Hospital (RML) Hospital, New Delhi, India

Sir,

Olanzapine, an atypical antipsychotic agent, is not commonly associated with significant hyperprolactinemia due to its weak dopamine binding capacity.[1] Previous reports suggested that olanzapine might be a safe alternative treatment for cases with antipsychotic induced hyperprolactinemia.[2,3] It has little effect on prolactin levels and is generally regarded to be prolactin sparing, although at higher doses hyperprolactinemia may result.[4,5] Contrary to this, we wish to report a case of olanzapine-induced breast enlargement secondary to hyperprolactinemia in a woman with obsessive compulsive disorder (OCD) at a low dose of 5 mg/day.

A 40-year-old married female presented with a history suggestive of OCD for last 20 years. She was treated with a number of antiobsessive drugs in adequate doses and for adequate duration but with minimal response. Her past and family histories were not significant. Personal history revealed interpersonal problems with husband and her in-laws. The patient was started on a combination of clomipramine and sertraline. Clomipramine was increased to a dosage of 275 mg/day and sertraline to 200 mg/day. However, with this combination, the patient did not show significant clinical response. The compliance was adequately ensured and the combination was continued for 4 months at the same dosage. Simultaneously, behavior therapy in the form of exposure and response prevention was started. The patient, however, did not cooperate for behavior therapy as, at times, there was no co-therapist available, and on other occasions, the patient did not comply with the instructions given. As a result, olanzapine at a dose of 5 mg/day was added to the existing treatment. After about 2 months of addition of olanzapine, the patient came for follow-up with complaints of bilateral enlargement of the breasts. The enlargement was diffuse, symmetrical, non tender without galactorrhea. She also had amenorrhea for 2 months. Investigations revealed serum prolactin level increased to 97 ng/mL (normal range = 1.5–19.0 ng/mL). Magnetic resonance imaging of the brain did not show any evidence of microadenoma. Other hematological investigations like complete blood count, liver function tests, blood urea, serum creatinine, glucose tolerance test and thyroid function tests (T3, T4 and TSH) were within normal limits. Her pregnancy test was negative. There was no other significant finding on general physical or systemic examination. There was no family history of breast related disorders. Olanzapine was stopped and patient was monitored for the next 1 month. Serum prolactin level estimated after 1 month of drug discontinuation was 25 ng/mL. Her menstrual cycles regularized after 2 months but there was no improvement in her breast enlargement. Subsequently, the patient was referred to surgery department where breast reduction surgery was performed. The breast tissue removed weighed around 2.5 kg and its histopathology revealed hypertrophy of glandular tissue. Her prolactin level was checked again after 2 months of surgery and was 10 ng/mL. Buspirone at a dose of 30 mg/day was added and the patient showed some improvement in her symptoms with addition of buspirone to a combination of clomipramine and sertraline.

In view of the temporal correlation between the initiation of olanzapine and development of breast enlargement and elevation of prolactin levels and return to normal prolactin levels subsequent to olanzapine withdrawal, olanzapine seems to be the likely causative agent. The score on Naranjo adverse drug scale was 6, suggesting a probable relationship with the drug.[6]

Other causes of hyperprolactinemia like pregnancy, thyroid disorders, acromegaly, pituitary microadenoma, etc.[7] were ruled out on the basis of normal investigations and no abnormal findings on general and systemic examination. The histopathological examination of the breast tissue also did not reveal any other pathology.

Olanzapine has been tried for augmentation in patients with OCD.[8] However, peculiarities of our case included the fact that the patient was only on 5 mg/day of olanzapine and that the patient had to undergo a major operation for the side effect which was distressing to the patient. Our report suggests that one should be cautious while prescribing antipsychotic agents to non psychotic patients who might be more sensitive to the side effects of these medications. Also, to the best of our knowledge, the literature search did not reveal any report of bilateral breast enlargement due to hyperprolactinemia with olanzapine. Bilateral breast enlargement as a consequence of hyperprlactinemia has not been reported.[9,10] We hope our clinical experience with olanzapine stimulates additional systematic clinical trials to reveal the magnitude of such side effects.
